# A Large N400 but No BOLD Effect – Comparing Source Activations of Semantic Priming in Simultaneous EEG-fMRI

**DOI:** 10.1371/journal.pone.0084029

**Published:** 2013-12-31

**Authors:** Sebastian Geukes, René J. Huster, Andreas Wollbrink, Markus Junghöfer, Pienie Zwitserlood, Christian Dobel

**Affiliations:** 1 Institute for Psychology, Westfälische Wilhelms-Universität, Münster, Germany; 2 Experimental Psychology Lab, Department of Psychology, Carl-von-Ossietzky Universität, Oldenburg, Germany; 3 Institute for Biomagnetism and Biosignalanalysis, Medical Faculty, Westfälische Wilhelms-Universität, Münster, Germany; University of British Columbia, Canada

## Abstract

Numerous studies have reported neurophysiological effects of semantic priming in electroencephalography (EEG) and in functional magnetic resonance imaging (fMRI). Because of differing methodological constraints, the comparability of the observed effects remains unclear. To directly compare EEG and fMRI effects and neural sources of semantic priming, we conducted a semantic word-picture priming experiment while measuring EEG and fMRI simultaneously. The visually presented primes were pseudowords, words unrelated to the target, semantically related words and the identical names of the target. Distributed source analysis of the event-related potentials (ERPs) successfully revealed a large effect of semantic prime-target relatedness (the N400 effect), which was driven by activations in a left-temporal source region. However, no significantly differing activations between priming conditions were found in the fMRI data. Our results support the notion that, for joint interpretations of existing EEG and fMRI studies of semantic priming, we need to fully appreciate the respective methodological limitations. Second, they show that simultaneous EEG-fMRI, including ERP source localization, is a feasible and promising methodological advancement for the investigation of higher-cognitive processes. Third, they substantiate the finding that, compared to fMRI, ERPs are often more sensitive to subtle cognitive effects.

## Introduction

The term *semantic priming* describes the facilitated processing of a meaningful stimulus when it is accompanied by another stimulus related in meaning [1]. Semantic priming is a cognitive phenomenon that is well established behaviorally [2], [3], with electroencephalography (EEG) [4] and, to a lesser degree, with functional magnetic resonance imaging (fMRI) [5], [6]. Most prominently, the discovery of the N400 effect [7], an event-related potential (ERP) component in the EEG closely related to semantic priming, has greatly contributed to the understanding of the effect's determinants and its functional significance. In recent years, and using a wide array of methodological approaches, increased effort has been spent to identify neural sources of the behavioral semantic priming effect and of the N400 ERP response [5], [6].

The number and location of identified brain regions potentially related to semantic priming varies between studies, materials, tasks and methods, but some converging evidence exists. Most notably, studies with lesion data (e.g., [8]), intracranial EEG recordings (e.g., [9]), localization based on magnetoencephalography (MEG) [10]–[12] and fMRI [13]–[16] all point to a broad region encompassing the left superior and middle temporal gyri (STG/MTG) as a major generator of the neural priming response.

While the methods mentioned give complementary insights into the neural basis of semantic priming, they differ substantially in terms of their experimental procedures, participant samples and, in particular, with respect to the aspects of neural processing they reveal best. Therefore, it remains challenging to put the results from these different methods into perspective, and, consequently, to obtain a complete picture of the underlying neural dynamics.

One way to overcome some of these differences is to use the identical experimental procedure and the same participants in two successive measures (as was done, for example, by Matsumoto et al. [16]). But even under such conditions, the presentation of the stimulus material has to be repeated, and internal and external factors may change between the sessions (e.g., baseline brain activity, psychological and physiological [e.g., emotional, attentional, hormonal] state of the participant, body posture of the participant, MR scanner noise [17]). As a result, comparisons of the two datasets are still inherently limited.

The recently developed possibility to record EEG and fMRI data simultaneously promises to minimize the above problems. In this method, the recorded data are in response to identical experimental stimulation, recorded in the identical environment, and they are based on identical underlying brain activity of the same participant [18]–[20]. Accordingly, the data from EEG and fMRI may be directly compared, and we can now exclude that differences between experimental effects in the two measures are caused by repetition and order effects, or by one of the internal or external factors listed above.

In the present study, we measured EEG and fMRI simultaneously in the context of a semantic priming experiment. For data analysis, we decided to directly compare experimental effects and source localizations between the two neurophysiological measures. We consider such a direct comparison to be valuable in and of itself, because it offers the much-needed opportunity to contrast EEG and fMRI activations to identical brain activity. Moreover, such a comparison represents a thorough empirical starting point to link the existing EEG and fMRI literatures.

In the study reported here, we implemented a priming design with words as primes and pictures as targets. There is ample evidence for priming effects in EEG [21] and MEG [11], [12] with words priming responses to pictures. Our particular rationale was to validate N400 localization results from our previous MEG experiments [11], [12] by applying distributed source localization to the EEG data recorded in the MR scanner. In addition, we measured fMRI (Blood Oxygen Level Dependent, BOLD) activations evoked by the identical experimental stimulation, with the purpose to replicate existing results from the semantic priming literature, and to compare the outcome with the EEG effects. We conducted a semantic priming experiment that was adapted from two of our previous studies, and that is known to produce a large N400 effect sensitive to the semantic similarity (or distance) between primes and targets [12], [22]. Participants performed a semantic classification task on pictured objects that were preceded by visually presented word primes. Primes were the identical names of the objects, semantically related words, semantically unrelated words or pseudowords. We predicted that the semantic-priming related electrophysiological and hemodynamic correlates would show increased activation as a function of the semantic distance between primes and targets. We also predicted that the underlying neural sources of the semantic priming effect in EEG and fMRI would overlap in the left temporal lobe.

## Materials and Methods

### Participants

Fifteen right-handed native speakers of German participated in the study (9 female, mean age  = 26.4 years, range  = 22 to 29 years). All had normal or corrected-to-normal vision. One participant's dataset had to be discarded from the EEG analysis and four from the fMRI analysis due to excessive artifacts or technical problems during data acquisition.

### Ethics Statement

Participants were informed about the risks, aims and procedure of the study and gave their written consent. The study was approved by the ethical committee of the German Society for Psychology (DGPs).

### Materials

Based on a previous study [22], 38 pictures were chosen as targets for the experiment, half depicting man-made objects, half nature-made objects. The pictures were selected from a photo database (Hemera Photo Objects, Hemera Technologies, Gatineau, Canada).

**Figure 1:**
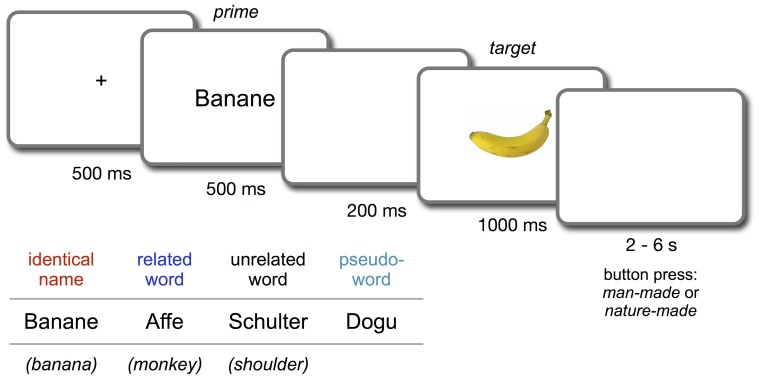
Illustration of the trial time course in the semantic priming paradigm. Example primes for the four conditions are presented at the bottom left, with English translations in parentheses. Participants were instructed to press the button only after the disappearance of the target picture, i.e., during presentation of the following blank screen.

Four primes (one from each condition) were selected for each picture. For three of the four priming conditions, 38 word primes each were selected from the earlier study [22]. These words were either *identical names* of the targets, *semantically related* words or *semantically unrelated* words. The fourth priming condition consisted of 38 *pseudoword* primes taken from another study [23]. Related primes had been selected based on the Edinburgh Association Thesaurus [24] and had then been rated by 22 subjects for semantic closeness to their targets on a 7-point scale (1 =  *not related at all*; 7 =  *strongly related*). The mean relatedness score for semantically related prime words was 6.3 (*SD* = 0.2). Semantically unrelated primes had a mean relatedness of 1.4 (*SD* = 0.3) (see Fig. 1 for an example). Pseudowords were four-letter words pronounceable to German speakers that did not systematically evoke particular associations, and were neutral with respect to their perceived valence [23].

### Semantic priming procedure

Word and picture stimuli were presented centrally on a white background using Presentation 12.2 experimental software (Neurobehavioral Systems, San Francisco, California) and an MR suitable projection system (Sharp XG-PC10XE). The eye-to-screen distance was 105 cm and all stimuli had a maximum viewing angle of 6° in height and width. Words appeared in black “Arial” font, with first letters capitalized (coherent with German orthography for nouns). Participants gave button responses with their right hand's middle and index fingers on an MR-compatible response pad.

Figure 1 illustrates the time course of a single trial. Each trial began with the presentation of a fixation cross for 500 ms followed by the prime word, which lasted for 500 ms. After a blank screen of 200 ms the target picture was presented for 1000 ms. The prime-target stimulus-onset asynchrony (SOA) was 700 ms. Participants had to classify the target picture as man-made or nature-made by pressing one of two buttons while ignoring the prime. Button assignments were counterbalanced across participants. No feedback was given. To avoid readiness potentials and movement artifacts due to reaction preparation, participants were instructed to perform the button press only after the picture had disappeared, thus rendering reaction times uninteresting. The word primes for every target were members of the same response-relevant category as the target itself (man-made or nature-made), to avoid confounds in the semantic categorization task due to incongruence of prime and target. A variable interval of a random length between 2 and 6 seconds was inserted between the disappearance of the picture and the appearance of the fixation cross of the following trial. The mean trial length was 6.2 s. Trial initiation was triggered by the fMRI clock. This was done to ensure that, throughout the experiment, event onsets for every condition were equally distributed across the 3 second time interval used for one fMRI volume acquisition (repetition time).

All 38 target pictures were pseudorandomly presented four times in each of two consecutive runs (38×4×2 = 304 trials), once with each of its four prime words. The order of presentation was constrained to allow no more than three consecutive trials from the same condition and to contain an even distribution of the possible transitions between the conditions of two consecutive trials.

### Simultaneous EEG-fMRI recording

#### EEG recording and parameterization

Simultaneously with fMRI acquisition, the EEG was recorded using an MR compatible amplifier (BrainAmp MR plus, Brain Products, Gilching, Germany). A 32-channel EEG cap (model “Easycap BrainCap-MR 3-0 32Ch) with 30 scalp electrodes located according to the international 10-10 system was employed. Of the two remaining electrodes, one was positioned on the back, left of the spinal column (between the 5^th^ and 7^th^ costa), to record the electrocardiogram, and one was positioned under the left eye to record the electrooculogram. To avoid uncomfortable pressure due to resting on the electrode cap and to avoid increased head movements, the participants' heads were rested on custom foam padding that included holes for electrodes and cables. During measurement, electrode impedances were kept below 15 kΩ, with FCz serving as the reference channel. The hardware clock of the EEG amplifier system was synchronized with the clock driving the MRI scanner's gradient switching system by means of a commercial device (SyncBox, Brain Products, Gilching, Germany), to guarantee the temporal stability of the EEG acquisition in relation to the switching of the gradients during the MR acquisition. The data were recorded with a pass band of 0.016–250 Hz and digitized at 5000 Hz at 16 bit with 0.5 µV resolution (dynamic range, 16.38 mV).

In a first step, EEG data were corrected for MR gradient and ballistocardiac artifacts by applying modified versions of the averaged artifact subtraction and adaptive noise cancellation algorithms proposed by Allen and colleagues [25], [26], implemented in VisionAnalyzer 1.05 (BrainProducts, Gilching, Germany). The MR-denoised EEG data were down-sampled to 250 Hz, re-referenced to an average reference and filtered with a pass band from 0.5 Hz to 70 Hz. Data were visually screened for gross non-stereotypic artifacts and affected intervals were excluded from further analysis. This was done because the to-be-performed artifact correction procedure based on independent component analysis (ICA) is best-suited only for repetitive, uniform artifacts such as eye blinks and line noise [27].

Time-locked to the picture onset, segments of 1200 ms length were then extracted from the data. Segments began 200 ms before the picture onset and lasted until 1000 ms after picture onset. Baseline correction was performed using the 200 ms interval preceding the picture.

To further reduce MR-related and common EEG artifacts, we applied extended infomax ICA [28], [29] on each participant's set of segmented data. Independent components that clearly corresponded to blinks, horizontal eye movements as well as components that could be assigned to residual gradient and ballistocardiac artifacts were manually identified and rejected. After backprojection of the remaining independent components, the EEG data were restored in their original time and voltage scale. Finally, any segments that included potentials exceeding a 70 µV threshold were discarded.

ERPs were then calculated for each experimental condition of each participant's data set. The number of trials surviving artifact rejection and correction (*M* = 68.61 of 76 trials per condition) did not differ significantly between conditions, *F*(3, 39) = 1.89, *p* = .148.

#### FMRI protocol and image preprocessing

Magnetic resonance imaging was performed at 3 Tesla on a Philips Medical system (Best, the Netherlands) equipped with a standard birdcage head coil, at the Institute for Clinical Radiology, University of Münster, Germany. An extended series of echo-planar-images (EPI) were gathered, including whole-head measurements before and after the experimental task. Thirty-six axial slices were obtained aligned to the anterior and posterior commissure (thickness 3.6 mm), using a multi-slice EPI sequence with an echo time of 38 ms, a flip angle of 90° and a repetition time of 3000 ms. The fMRI pixel matrix acquired was 64×64, with a field of view of 230×230 mm^2^, resulting in a voxel resolution of 3.6×3.6×3.6 mm^3^. The same parameters were also used to cover the whole brain with 43 slices. These whole-head EPIs were later used to optimize the spatial normalization of the functional echo planar images.

The basic preprocessing of the images and fMRI-statistics were done using SPM5 (www.fil.ion.ucl.ac.uk/spm). After slice-time correction, spatial realignment and co-registration of the functional images to the whole head EPIs, all images were normalized to match the Montréal Neurological Institute EPI-template provided by SPM5. Images were resliced to isometric voxels with an edge length of 2 mm in each direction. Smoothing the images with an 8 mm FWHM-kernel finished the preprocessing steps.

### Statistical analysis of fMRI and EEG data

#### Analysis of EEG source activations

**Figure 2:**
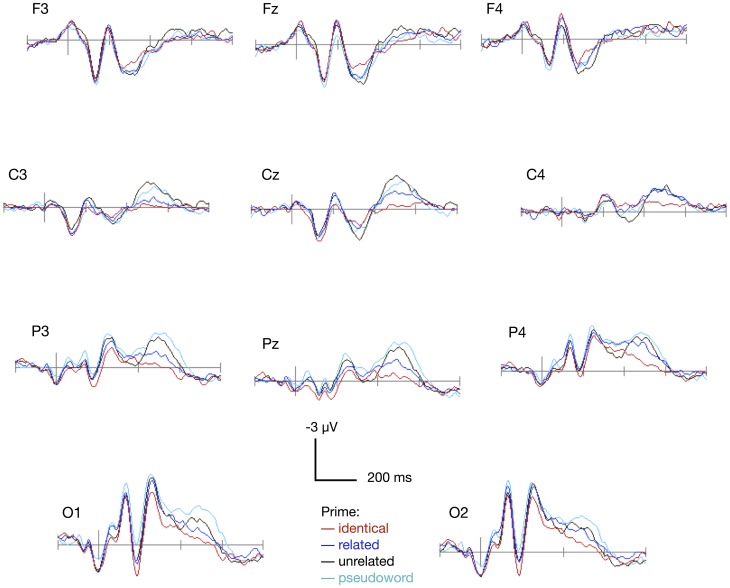
Grand-averaged event-related potentials. ERPs from eleven standard electrode positions are presented, plotted by priming condition in an interval stretching from 200

Because we were interested in the generators of neural activity itself, statistical analyses of ERP data will be presented in source space activity estimates only. Notwithstanding, as shown in Figure 2, sensor space waveforms revealed the expected topography and qualitatively similar effects in terms of condition differences as the source waveforms.

To retain comparability with previous localization studies [11], [12], [30]–[32], ERP source analysis was based on a spherical head model. ERP source space activity was analyzed using MATLAB-based ElectroMagneto-Encephalography software (EMEGS, version 2.4, www.emegs.org, [33]). Source space activity was estimated for each time point in each condition and participant, using the least square minimum norm estimation (L2-MNE) method [34]. This inverse modeling was based on a four-shell spherical head model with evenly distributed 3 (radial, azimuthal and polar direction) ×350 dipoles as source model [35]. A source shell radius of 87% of the spherical volume conductor head radius was chosen, roughly corresponding to the grey matter volume. Across all participants and conditions, a Tikhonov regularization parameter k of 0.05 was applied [33]. We also calculated solutions with a k value of 0.01, 0.02, and 0.2. They showed qualitatively very similar results, indicating that the parameter choice did not bias our interpretation of ERP activations.

To accurately assess the sources and time interval(s) relevant to the semantic priming effect whilst avoiding circular analysis, the data were analyzed in a two-level procedure, in accordance with the independent split-data approach [36]. The participant sample available for ERP data analysis was randomly split into two groups of 7 participants. Data from the first half of participants (referred to as sample *n*_1_) were used for time interval and region of interest (ROI) selection, based on pointwise statistics. No further EEG analysis was performed on this half of participants. Using the determined interval of interest and the ROIs, mean L2-MNE activations were then extracted from the data of the second half of participants (sample *n*_2_). These mean activations were entered into a repeated-measurement ANOVA to test for the predicted linear effect among semantic priming conditions and to assess the lateralization of the priming effect.

#### Analysis of fMRI activations

For each subject, a fixed-effects analysis was applied, using a canonical hemodynamic response function (HRF) to model the four priming conditions (identical name, related word, unrelated word, pseudoword). The events for each of the four conditions were modeled by specifying the onset of the target stimulus in time and considering the events instantaneous (SPM parameter 0). In addition, movement-related parameters derived from the spatial realignment were included as covariates to further control for task-irrelevant artifacts (movements did not exceed those typically observed in fMRI-only measurements). Contrast maps of the four experimental conditions of each subject were entered into a random-effects group analysis based on the General Linear Model as implemented in SPM5. A conjoined *F*-statistic as well as separate paired *t*-tests against the implicit baseline were computed for the priming conditions, thus testing for common as well as distinct patterns of activation across the experimental conditions. A family-wise error correction at the level of *p*≤.05 was applied for all whole-head fMRI-statistics reported.

## Results

### Behavioral results

Participants were instructed to give responses (man or nature made) only after the target picture had disappeared. Therefore, reaction time data were not analyzed. The error rate was low and did not differ significantly across conditions, *M* = 2.68, *F*(3, 39) = 0.94, *p* = .427.

### EEG data

**Figure 3:**
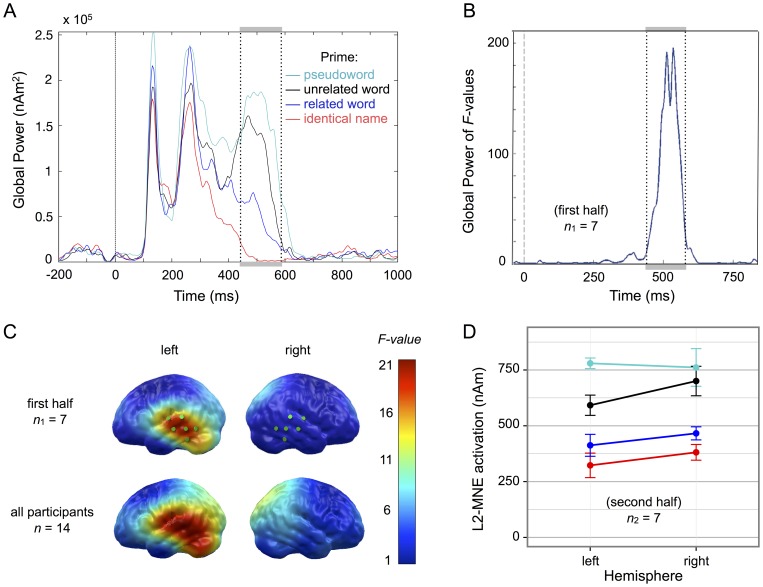
Results from the EEG source analysis. a) Global Power of estimated EEG source activations across all test dipoles. b) Global Power of *F*-values for the main effect *Semantic Relation* from a pointwise repeated-measurement ANOVA (based on half the participant sample). The interval of interest selected for second-order analysis is indicated by dotted lines. c) Statistical parametric maps (SPMs) of the *F*-values for the main effect of *Semantic Relation* from the same pointwise repeated measurement ANOVA (mean *F*-value in the interval of 440 – 590 ms after picture onset, projected onto a standard cortical surface). The upper row shows SPMs for the first half of participants, on which selection of the interval and ROIs was based. Dipole clusters selected for second-order analysis are marked in green. For comparison, the lower row shows SPMs for the entire set of participants. d) Mean L2-MNE source activations from the second half of participants for the selected interval and ROIs, plotted by priming condition [colors as in a)]. Error bars represent within-participant standard errors following Cousineau [59].

The Global Power of the estimated neural activities across all test sources is shown in panel A of Figure 3. Differential EEG source activations for the four semantic priming conditions can be seen in the interval about 400 to 600 ms after picture onset, corresponding with the pattern visible in the sensor data (Fig. 2). As predicted, the mean Global Power within this interval decreased from the pseudoword condition, to the unrelated word condition, to the related word condition, and finally to the identical name condition.

To select the interval for second-order analysis, we opted for a data-driven approach: a pointwise one-way repeated measurement analysis of variance (ANOVA) with the 4-level factor *Semantic Relation* (prime  =  identical name, related word, unrelated word, or pseudoword) was conducted on the Global Power values from the first half of participants (*n*_1_ = 7). Based on the Global Power of the *F*-values from that pointwise ANOVA (cf. Fig. 3, panel B), an interval of interest for the semantic priming effect was identified defined as the interval that included values larger than 16, spanning from 440 to 590 ms. No further intervals indicated a significant effect of priming. To assess the experimental effect in more detail, the sources most strongly involved in the semantic priming effect were investigated using second-level statistics. For this purpose, with the same data from sample *n*_1_ and based on the selected interval, mean source activations were computed and a one-way repeated measurement ANOVA with the factor *Semantic Relation* was calculated for each test dipole. The region of interest for second-order statistics was selected based on the resulting *F-*statistics: A single dipole cluster of six neighboring left-temporal dipoles from the outer shell distinctly showed the strongest priming effect, with each dipole showing a main effect of *F*(3, 18) >18. The cluster was therefore included as a ROI (cf. Fig. 3, panel C). To test for lateralization effects, the cluster of the six lateral symmetric right-hemispheric dipoles was added as a second ROI.

Data from the second half of participants (*n*_2_ = 7) were then used for second-order analysis. Mean L2-MNE source activations for each condition were calculated based on the independently determined interval and ROIs. Within both of these ROIs, the pseudoword condition again showed the highest source amplitude, followed by the unrelated condition, the related condition and the identical name condition (Fig. 3, panel D). A repeated-measurement ANOVA with the factors *Semantic Relation* and *Hemisphere* indicated a significant main effect of *Semantic Relation, F*(3, 18) = 18.99, *p* <.001, η_p_^2^ = .76, but no main effect of *Hemisphere, F*(1, 6) = 1.00, *p* = .356, η_p_^2^ = .14, and no interaction between the two factors, *F*(3, 18) = 1.77, *p* = .189, η_p_^2^ = .23; all reported *p*-values are Greenhouse-Geisser corrected if required. In accordance with our prediction, the main effect of *Semantic Relation* was best described as a linear trend, *F*(1, 6) = 25.13, *p* = .002, η_p_^2^ = .81. In a tentative analysis using the whole participant sample (*n* = 14), the interaction of *Semantic Relation* and *Hemisphere* did indeed reach significance, *F*(3, 39) = 3.87, *p* = .037, η_p_^2^ = .23, indicating that a larger differentiation of priming conditions is present in the left hemisphere. To obtain full comparability with fMRI activations, the analysis on sample *n*_2_ was also re-run while excluding data from the one participant that did not enter fMRI analysis. The pattern of results remained identical: main effect *Semantic Relation*, *F*(3, 15) = 15.01, *p* = .002, η_p_^2^ = .75, main effect *Hemisphere*, *F*(1, 5) = 0.04, *p* = .850, η_p_^2^ = .01, interaction of *Semantic Relation* by *Hemisphere, F*(3, 15) = 1.42, *p* = .288, η_p_^2^ = .22.

### fMRI data

**Figure 4:**
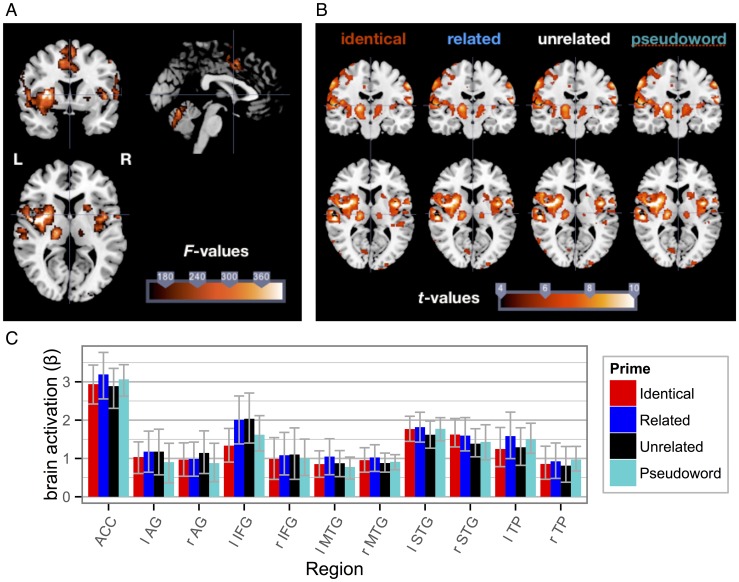
Results from the fMRI analysis. a) Coronal, sagittal and axial view for the whole-head contrast of the effect of stimulus presentation against the implicit baseline. b) Whole-head contrasts of the individual priming conditions against the implicit baseline in coronal and axial views (visualizations correspond to *p*<.0001 and below). c) Mean activations and bootstrapped 95% confidence intervals for selected brain regions, plotted by condition. Note: l = left, r = right, ACC = anterior cingulate gyrus, AG = angular gyrus, IFG = inferior frontal gyrus, MTG = middle temporal gyrus, STG = superior temporal gyrus, TP = temporal pole.

Statistical parametric maps from the random effects group analysis of fMRI data are depicted in Figure 4. Common patterns of activation across conditions are shown in panel A, based on the *F*-statistic from the General Linear Model. Contrast maps of the individual priming conditions against the implicit baseline (zero) are depicted in panel B, with *t*-values indicating the strength of an effect. Salient clusters of activity were found in left lateral temporal, bilateral medial-temporal sites (insula, putamen), bilateral dorsal anterior cingulate cortex (ACC) and adjacent bilateral (pre-) supplementary motor areas (pre-SMA). A detailed list of significant activations can be found in Table 1.

**Table 1:** Brain regions showing increased activation following stimulus processing.

				Cluster size	MNI coordinates		
H	Anatomical region	BA	*t-*max	(voxels)	x	Y	z
L	post-central	4	18.91	497	−60	−8	46
L	superior temporal gyrus	22	29.89	1907	−58	−22	9
L	insula	48	38.35	1613	−44	−2	0
L	putamen	48	22.67	1103	−30	4	8
L	cerebellum	18/19	20.49	511	−20	−86	−20
B	(pre-) supplementary motor area	6	23.10	616	−6	0	60
B	dorsal anterior cingulate	24/32	23.22	615	10	16	36
R	insula	48	24.39	314	42	−2	10
R	putamen	48	19.62	311	22	14	4
R	cerebellum	18/19	31.32	2087	18	−58	−22

*n* = 11; H = hemisphere, L = Left, B = Bilateral, R = Right, BA = Brodman area, *t*-max = maximum *t*-value within the cluster.

When comparing the individual conditions using fMRI, it seemed that activation patterns were virtually identical to the overall effect. Indeed, statistically comparing the pseudoword against the three word priming conditions (pseudoword – unrelated, related, and identical, respectively) by means of *F*- and *t*-contrasts, did not reveal any significant differences between conditions with the whole-head analyses (testing against *p* <0.05 and applying a family-wise error correction).

### Exploratory analyses

Two steps were taken to further address whether priming-related differences corresponding to those seen with the EEG might simply be less pronounced yet still present in the fMRI data. First, whole-head fMRI *F*-contrasts comparing the pseudoword to the other priming conditions (identical, related, unrelated) were computed at *p*<0.05 without correcting for multiple-comparisons. Only then, small clusters of differential activations emerged that also included bilateral mid-temporal as well as inferior frontal regions. Then, mean activations for each of the four conditions were computed in volumes of interest (VOIs) that have been associated with semantic priming in the literature (bilateral IFG, bilateral MTG, bilateral STG, bilateral temporal poles, bilateral angular gyri, ACC [5], [16]), which also partially coincided with the exploratory whole-head analyses. Masks for the extraction of the mean from condition-specific beta images were derived from the AAL toolbox [37]. Regionally specific mean activations were analyzed by means of a repeated-measures ANOVA computed for each of the VOIs, again testing against *p*<0.05 without correcting for multiple-comparisons. Mean activations and corresponding confidence intervals are shown in Figure 4 C. No priming effect was found within any of the VOIs (all *F*s<1.89, all *p*s>.20).

Furthermore, to investigate the relationship between the fMRI and ERP activations, we calculated correlations of the experimental effect in the identified ERP sources with those from selected fMRI clusters, similar to the approach taken by Matsumoto and colleagues [16]. Following their procedure, we calculated the differences between mean activations to unrelated vs. related primes (semantic effect), and additionally, we calculated the unrelated vs. identical difference (identity effect). Also similarly, the number of participants for which both EEG and fMRI data were available was small (*n* = 10), such that the following results should be interpreted with caution. Because our aim was to test whether the fMRI activations were at all related to the identified (semantic and identity) ERP effects, and to keep the number of statistical tests small, we decided to base correlations on the supposedly strongest individual ERP effects, i.e. those in the identified temporal regions. These ERP effects were thus defined as the difference of the respective conditions' mean activations (unrelated – related, unrelated – identical) in the above-identified bilateral temporal clusters and in the selected (440 − 590 ms) time interval, averaged over both hemispheres (i.e., corresponding to the data from Figure 3 D, but now drawn from the 10 participant sample that was available here). These difference values were then correlated with corresponding fMRI activation differences in the left and right IFG, the left and right MTG, and the ACC. We report one-sided *p-*values because we expected the effects to correlate positively. The reported *p*-values are not corrected for multiple comparisons. For the semantic effect (unrelated - related), no significant correlations were found (all *r*s<.42, all *p*s>.115). However, for the identity effect (unrelated – identical), the left MTG, *r*(8) = .65, *p* = .021, and both hemispheres' IFGs showed significant correlations under these liberal criteria, left IFG, *r*(8) = .73, *p* = .008, right IFG, *r*(8) = .67, *p* = .017; the right MTG and the ACC did not: right MTG, *r*(8) = .37, *p* =  = .146, ACC, *r*(8) = .20, *p* = .292.

## Discussion

In the present study, we simultaneously recorded EEG and fMRI during a semantic word-picture priming experiment to compare experimental effects and neural sources from these two measures. We performed distributed source analysis on the ERP data using L2 minimum norm estimation, and compared the results to fMRI data that were analyzed using statistical parametric maps derived from a general linear model based on the canonical HRF.

As predicted, EEG source activity during the N400 interval was strongly modulated by the semantic similarity or distance between prime words and target pictures: The larger the semantic distance, the larger the N400 amplitude. This gradation of N400 amplitudes along semantic distance corresponds to previous results from EEG and MEG studies, with pictures and with words as targets [12], [38]. The notable size and temporal selectivity of the observed effect leads us to conclude that the employed paradigm elicited the N400 response as intended.

Distributed source analysis of the ERP data based on the L2 minimum norm procedure revealed that the sources showing the strongest priming effect were situated in a confined region of the left temporal lobe. Second-level analysis within this left-temporal and a contralateral cluster showed that the differentiation of N400 source amplitudes among priming conditions followed the predicted order in a linear fashion. The observed ERP localization corresponds with the general pattern from previous MEG and EEG studies on semantic priming that were performed outside the MR scanner [11], [12], [39], [40]. Given that EEG signals recorded simultaneously with fMRI can be severely degraded [19] and considering that the number and location of identified N400 sources may vary substantially between studies and individual subjects [6], [38], the clarity of the present result is remarkable.

In the fMRI data, whole-brain analysis revealed widespread *general* activation increases following stimulus processing as compared to the implicit baseline, overlapping with identified ERP N400 sources in the lateral part of the left temporal lobe. Contrary to our prediction, no *differential* activations between the priming conditions were found with conventional significance testing. This absence of differential priming effects in BOLD activations was confirmed in post-hoc volume-of-interest analyses, focusing on brain regions that have been associated with semantic priming in fMRI. Because the observed ERP N400 effect was large in amplitude and extremely stable across participants, and because stimulus presentation was followed by substantial general BOLD signal increases, we are confident that neither a suboptimal experimental design nor a lack of power is responsible for the null effect in the BOLD condition contrasts. Nevertheless, we further calculated exploratory analyses for BOLD differences between conditions omitting corrections for multiple testing, and we also computed correlations between the (semantic and identity) priming effects from selected ERP and fMRI sources. Results indicated that the identity effect (unrelated – identical) in the ERP correlated with the corresponding effects in the left MTG and in both hemispheres' IFGs, while this was not the case for the right MTG and the ACC. Thus, despite the absence of statistically significant BOLD priming effects at the group level, individual BOLD effects in the left MTG and the bilateral IFGs still show some association with individual ERP effects. In sum however, these data suggest a strong discrepancy between EEG and fMRI regarding the sensitivity to priming-related changes in neural activity. Considering data from fMRI studies on semantic priming and our own large ERP effect, this finding demands further discussion. We will first discuss the null effect in fMRI in the context of other studies, and we will then turn to the within-study discrepancy between the ERP and fMRI results.

The current study was designed to replicate the MEG effect from an earlier word-learning study [12] in ERPs recorded simultaneously with fMRI. It thus deviates in several design characteristics from other fMRI studies on semantic priming. One important difference concerns the target format. We used word primes and picture targets instead of the commonly used word targets, resulting in a “cross-format” design (here: words and pictures, both visually presented). In EEG, N400 effects for picture targets have been observed [41]–[43], and are not qualitatively or quantitatively very different from word-based responses. While there might be slight topographical differences depending on the stimulus format, there is certainly no reason to assume that the N400 effect is specific to a particular format or sensory modality [4]. Our previous cross-modal (spoken words and picture targets) MEG word-learning study [12] corroborated this with regard to neural generators: we observed the same left-temporal sources commonly found for word-based N400 effects to be also involved in the generation of picture-based N400 effects. Thus, the electrophysiological evidence does not suggest that large-scale changes to the fMRI response should be expected with picture targets or with a cross-modal / cross-format design.

With regard to the fMRI literature, there are several studies that used word-picture presentations in picture-naming paradigms [44]–[46]. However, we consider such production results not to be readily comparable to comprehension data. This is because the typical semantic effect in picture naming (semantic interference) is thought to be located at the lexical [47] or even phonological level [44], while the effect observed in comprehension experiments (semantic priming) takes place on the conceptual level [3]. With comprehension tasks, there is to our knowledge only one study that used word primes and picture targets, by Simons and colleagues [48], who investigated whether the processing of an object picture changed depending on whether the object's name or a pseudoword was presented simultaneously. They found that a small region in the left fusiform gyrus was indeed sensitive to the identical name vs. pseudoword manipulation. But as this is the only study we are aware of, there seems to be very little published evidence on whether word-picture priming leads to reliable regional activations, and if so, whether these are independent of the target's format (that is, whether they overlap with activations identified in word-word priming). Possibly, the cross-format nature of the paradigm by itself may be a reason for the lack of priming effects in fMRI activations, although this seems at odds with what is observed in the EEG/MEG literature. In sum, given the lack of evidence and because cross-format priming is well-suited to specifically focus on the semantic aspect of the prime-target relation, further fMRI studies of the cross-format version of semantic priming are certainly desirable.

A further difference to previous fMRI studies concerns stimulus repetition. Our experimental design deliberately included repetitions of primes (2 presentations) and targets (8 presentations), while most fMRI studies were designed to avoid stimulus repetitions [16], [49]–[51]. Our earlier study [12] included only a limited number of targets because participants had to learn new names for these targets. To keep changes to the previous design small, we also employed a limited set of 38 targets, and repeated them in each priming condition within the same subjects. We also repeated the whole stimulus set twice to increase overall statistical power. We were of course aware of the fact that stimulus repetition typically leads to signal decreases. However, the previous study had shown clear N400 effects in MEG despite stimulus repetition, which is why we also used stimulus repetitions here. Additionally, because the targets here appeared equally often in all conditions, any differences between conditions cannot have arisen from target differences. Importantly, the present ERP results replicate our previous finding with MEG: a large N400 effect despite of stimulus repetition. For the fMRI data, repetition may have affected BOLD responses (as in repetition suppression, [52]). Although we feel that repetition suppression should not interact with the semantic manipulation, it is conceivable that repetition diminishes the chance to discover differences between priming conditions. Unfortunately, our design is not powerful enough to address this issue.

A third difference concerns response instruction. Most of the existing fMRI studies on semantic priming [14], [16], [49], [51] included a two alternatives forced-choice task, with participants giving speeded responses. In the current study, participants were instructed to delay their response until the picture had disappeared (1 s after stimulus onset), to avoid response activations in the ERP epochs. The behavioral priming effects observed in previous studies were very large (e.g. reaction time differences of 34 to 95 ms between unrelated and related conditions in the three experiments reported in Gold et al. [14]). Like the neurophysiological effect, these behavioral effects are driven by semantic priming (the very phenomenon was of course first described using reaction time effects [1]). It could well be that at least parts of the reported BOLD effects are driven by activity differences associated with speeded responding per se. It would therefore be of interest to assess more thoroughly whether particular fMRI correlates of semantic priming depend on the presence of speeded responding or, more generally, on overt responding per se. When interpreting the frequent left IFG activations to semantic priming, van Petten and Luka [6] have already hypothesized that the IFG might be involved in task-strategic processing that is independent of comprehension itself. They also suggested that it would thus be worthwhile to control for task-related effects in future studies. Somewhat related, Chen, Davis, Pulvermüller, and Hauk [53] have recently revealed a discrepant sensitivity of EEG/MEG and fMRI to task demands in single-word processing, further indicating that the nature of the task is particularly important in the context of EEG/fMRI comparisons. A systematic examination of task effects in the more extensive repetition priming literature might give some indication on the importance of task characteristics in the context of priming.

Taken together, design differences between the current study and existing studies on semantic priming make it difficult to single out one factor that may have led to the observed null result in fMRI. However, we think that these factors should be investigated more closely, such that we obtain a more generalized understanding of semantic priming and its neural correlates.

Independent of the differences between the current fMRI results and those from the literature, it is worth taking a closer look at the discrepancy between the fMRI and ERP effects within the current study. In their review, van Petten and Luka [6] discuss a number of reasons that could lead to differing ERP and fMRI effects and localizations of semantic priming – for example the potential uncertainty of fMRI source localizations as a consequence of blood vessel properties, the possibly overall lower signal-to-noise ratio of fMRI, and the phenomenon of event-related phase resetting as an example of a neurophysiological trace not detectable in fMRI. Because of existing evidence in the N400 context, we will briefly consider the latter explanation.

To address phase resetting in the context of the N400, Mormann et al. [54] analyzed intracranial ERPs recorded during a single-word processing experiment. Indeed they argued that phase resetting may be the driving factor underlying the N400 component. Mormann et al. [54] presented single words, some repeatedly, some not, to epilepsy patients undergoing surgery. Word repetition has been shown to cause a very similar N400 attenuation as the priming of a word by a related item [55], and both the repetition effect and the semantic priming effect are considered to indicate facilitated conceptual processing [4]. Mormann et al. [54] performed EEG depth recordings to measure the so-called AMTL-N400, an ERP component observed in the anterior medial temporal lobe (AMTL) that is considered to be closely related to the scalp-recorded component [9], [56]. In their analysis that distinguished between event-related power changes and event-related phase clustering, Mormann et al. [54] found that the AMTL-N400 amplitude difference between responses to repeated vs. novel words could not be explained by a difference in power. They rather suggested that it must be caused by a less variable timing of the EEG signal following new words (i.e. phase clustering), which in turn results in an amplitude difference in the averaged signal. They concluded that “verbal novelty in comparison with verbal recognition is associated with a reduced variability of the timing of stimulus processing, but not with an increased recruitment of neural assemblies within the rhinal cortex” ([54], p.898). If their analysis is valid and if we assume it to be similarly relevant in the scalp-recorded ERP component, then phase clustering could well explain why the probability of detecting an fMRI correlate that corresponds to the ERP effect is reduced in case of the N400 component.

Beyond this theoretical explanation, Vartiainen and colleagues [57] directly compared the neural responses from separate MEG and EEG/fMRI recordings during single-word reading. They observed that, apart from visual cortex, the localizations from MEG and fMRI differed markedly in the frontal and temporal cortex, including even opposite stimulus effects in some regions. Their results stress that the relationship between electrophysiological and hemodynamic measures of brain activity is far from straightforward. Recently, more advanced methods of fMRI analysis have emerged that might more aptly capture the complex neural networks involved in semantic processing (one interesting example is a pattern-classification fMRI study on word and picture stimuli that reference identical objects, by Shinkareva and colleagues [58]). Clearly, more research is needed to assess how electrophysiological, magnetencephalographic and hemodynamic responses differ, and simultaneous recordings are very important to this issue.

In conclusion, in this semantic word-picture priming experiment we obtained robust ERP effects in the N400 window, and the EEG source analysis clearly replicates findings of a predominantly left-lateralized temporal origin for these effects. We found little evidence that fMRI activations similarly distinguish between priming conditions. The fact that the EEG effect was considerable, despite of the simultaneous fMRI recording, underlines the usefulness of a combined registration for the investigation of higher cognitive processes, and their potentially different signatures in different brain measures.
